# The experiences of midwives with violence from patients, their relatives and friends: A study in perinatal center in Lithuania

**DOI:** 10.18332/ejm/215187

**Published:** 2026-02-28

**Authors:** Miglė Mikėnaitė, Mark Barakat, Bronislava Petrošienė, Mindaugas Kliučinskas, Arnoldas Bartusevičius, Alina Liepinaitienė, Eglė Bartusevičienė

**Affiliations:** 1 Department of Obstetrics and Gynecology, Lithuanian University of Health Sciences, Kaunas, Lithuania; 2 Department of Environmental Sciences, Faculty of Natural Sciences, Vytautas Magnus University, Kaunas, Lithuania; 3 Republican Šiauliai County Hospital, Šiauliai, Lithuania

**Keywords:** workplace violence, midwives, occupational health, healthcare professionals, verbal abuse

## Abstract

**INTRODUCTION:**

Workplace violence against healthcare professionals, particularly midwives, is a significant concern worldwide. Verbal violence, often underreported, can lead to emotional distress and impact professional performance. This study explores experiences of midwives with patient- and relative-related violence in a Lithuanian perinatal center.

**METHODS:**

A cross-sectional study was conducted from January to February 2022 at the Department of Obstetrics and Gynecology of the Lithuanian University of Health Sciences using a modified version of the ‘Violence in Nursing and Midwifery’ questionnaire. All midwives (n=90) working in this institution were invited to complete an anonymous questionnaire (response rate 80%, n=72). Primary outcomes were the frequency and types of workplace violence experienced by midwives. Secondary outcome measures included changes in midwives’ emotional and professional state, coping mechanisms and actions taken after an episode of violence.

**RESULTS:**

The majority (70.8%) of participants experienced or witnessed verbal violence, manifesting as unreasonable demands (72.5%), rudeness (66.7%), and anger (60.8%), with no physical violence reported. Emotional consequences included unhappiness (66.7%), anger (58.8%), irritability (41.2%), burnout (50.9%), and sleep disturbances (31.4%). The most effective actions in dealing with the psychological consequences of violence were informal debriefing with staff members (70.6%) and talking with friends and family after an episode (52.9%). Underreporting was common due to perceived ineffectiveness of long-term change, despite clear reporting procedures.

**CONCLUSIONS:**

Verbal workplace violence is widespread among midwives and contributes to psychological and professional distress. Strengthening organizational support, implementing targeted training programs, and establishing a national monitoring system are essential to address and prevent workplace violence in healthcare settings.

## INTRODUCTION

Violence as a public health issue was formally recognized in 1996 during the 49th World Health Assembly, which adopted a resolution declaring violence one of the most significant public health problems worldwide. This resolution highlighted the extensive consequences of violence on individuals, families, societies, and nations, highlighting its detrimental effects on health and an increased financial burden on healthcare systems^1^. Notably, non-physical violence is more prevalent than physical aggression, and certain occupations – particularly those in healthcare – are more closely associated with workplace violence^2^. According to World Health Organization (WHO), workplace violence is an incident where staff are abused, threatened, or assaulted in circumstances related to their work, involving an explicit or implicit challenge to their safety, well-being, or health^3^.

Among healthcare professionals, nurses are most at risk for patient-related violence, with 60–90% reporting experiences of both verbal and physical abuse, particularly in high-risk departments such as psychiatry and emergency care^4^. Midwives also work in highly stressful environments, routinely managing traumatic births, complex perinatal complications and medical emergencies – factors that can increase their risk of workplace violence^5^. In a recent questionnaire by the International Federation of Gynecology and Obstetrics Committee on Women Facing Crises, 70.5% of participating nurses and midwives reported experiencing violence while on duty. Abuse experienced by the midwife included physical and sexual assault, spitting, human waste attacks and verbal threats^6^. Workplace violence has a substantial negative impact on the quality of life and work productivity for healthcare professionals. Nurses who frequently encounter violent patients reported lower organizational commitment, poorer physical and mental health, and increased levels of frustration, anxiety, fear, and anger. In addition to short-term physical injuries, long-term health issues such as musculoskeletal disorders and post-traumatic stress disorder, such as insomnia, anxiety, nightmares and flashbacks, are commonly reported^7^.

In Turkey, a study found that 85.4% of midwives and nurses reported verbal violence and 34.3% experienced physical violence^8^. In response, nursing associations have advocated for anti-violence initiatives, such as questionnaires to gather data on workplace violence, as well as strategies to reduce violence, increase support, and promote prevention^9^. Reducing workplace violence has led to improvements in nurses’ quality of life, enhanced patient health outcomes, and even resulted in reduced healthcare costs^10^.

However, a comprehensive understanding of workplace violence can only be achieved by considering the perspectives of midwives and nurses on their experiences with patients, their relatives or friends related violence. This approach is essential to improve the management and reduce the frequency of such incidents.

Currently, Lithuania lacks a standardized national questionnaire to evaluate and monitor incidents of workplace violence experienced by midwives. The aim of this study is to explore midwives’ experiences with workplace violence at the University Hospital’s Department of Obstetrics and Gynecology, focusing on the frequency, types of violence, consequences of experiencing violence, and actions taken in response to such incidents.

## METHODS

### Study setting

The cross-sectional observational study was carried out from 1 January to 28 February 2022 at the Department of Obstetrics and Gynecology of the Lithuanian University of Health Sciences. It is a referral University hospital and one of two perinatal centers in Lithuania that provide specialized tertiary level care for pregnant and parturient women facing the most challenging pregnancies, childbirth and fetal health care issues across the country. All midwives (n=90) working in this institution were invited to complete an anonymous paper-based questionnaire on a voluntary basis. The exclusion criteria were as follows: midwifery students in training, less than six months’ work experience, and midwives who did not agree to take part in the survey. According to official records, in January 2022, 996 midwives held a midwife license in Lithuania. However, only about two-thirds of them were actively practicing (664 midwives). Given that our study included 90 midwives, this represents roughly 13.7 % of actively practicing population of midwives.

### Study instrument and outcomes

The ‘Violence in Nursing and Midwifery’ questionnaire of Pich^11^ was used as the main study instrument for gathering data. The questionnaire aimed to assess the frequency and types of violence experienced by midwives from patients and/or their families and friends, and to better understand consequences of violence, coping mechanisms and midwives’ perceptions of risk prevention. An English-Lithuanian translator translated the original questionnaire. All the linguistic and medical terms were discussed with an obstetrician-gynecologist and a midwife, fluent in English. Afterwards, the questionnaire was translated from Lithuanian back to English to prevent misapprehension.

An expert committee, consisting of a translator, obstetrician-gynecologists and midwives, evaluated the forward and backward translations. The questionnaire was slightly modified from the baseline questionnaire to better address midwives’ needs and was designed to be replicable on a national level. Questions that were not relevant for midwives, such as those concerning clinical areas of work and work location, were removed. Although the structure and content of the original questionnaire were not fully replicated, relevant issues and categorizations of verbal and physical violence were included. The adapted questionnaire consisted of five main sections: 1) General characteristics of the participants (age, work experience); 2) Incidence and characteristics of workplace violence (verbal or physical) in the past six months; 3) Consequences of experiencing violence (emotional and professional responses); 4) Coping mechanisms and actions taken after an episode of violence; and 5) Risk prevention strategies. At the beginning of the questionnaire, key terminology related to workplace violence, including physical violence and verbal violence, such as harassment, bullying, and threatening behavior, was defined. A six-month recall period was used to minimize bias in recalling past violent incidents.

The preliminary questionnaire was tested to assess the understanding of the questions and response options. Five native-speaking Lithuanian midwives who met the inclusion criteria filled in the questionnaire. They were encouraged to note any difficulties experienced when completing the questionnaire. After minimal corrections, the final version of the Lithuanian questionnaire was prepared.

The primary outcome measures were the frequency (percentage) and types of workplace violence experienced or witnessed by midwives. The secondary outcome measures included changes in midwives’ emotional and professional state resulting from exposure to workplace violence, coping mechanisms and actions taken after an episode of violence.

### Ethical considerations

This study was conducted in accordance with the Declaration of Helsinki. The approval of the Lithuanian University of Health Sciences Bioethics Center was received for this study [No. BEC-AK(B)-56].

### Data analysis

Statistical analysis was performed using the Statistical Package for the Social Sciences (SPSS), version 29.0.1 for Windows (Chicago, IL, USA). Categorical data are presented as frequencies and percentages. Categorical variables were analyzed using the χ^2^ test. A p<0.05 was considered statistically significant.

## RESULTS

### General characteristics of the participants

A total of 90 midwives filled in the questionnaire. Of these, 18 did not fully complete the questionnaire, resulting in 72 midwives (80%) being included in the study. Almost a third of midwives (30.6%) were aged 46–55 years, with 27.8% reporting work experience of 21 to 30 years (Table 1).

**Table 1:** Characteristics of participants in the cross-sectional study conducted at the Department of Obstetrics and Gynecology of the Lithuanian University of Health Sciences, January–February 2022 (N=72)

*Characteristics*	*Total* *(N=72)* *n (%)*	*Experienced workplace violence*		p*
		*No* *(N=21)* *n (%)*	*Yes* *(N=51)* *n (%)*	
**Age** (years)				
18–25	6 (8.3)	1 (4.8)	5 (9.8)	0.511
26–35	18 (25)	6 (28.6)	12 (23.5)	
36–45	19 (26.4)	8 (38.1)	11 (21.6)	
46–55	22 (30.6)	4 (19)	18 (35.3)	
56–65	7 (9.7)	2 (9.5)	5 (9.8)	
**Work experience** (years)				
<6	14 (19.4)	3 (14.3)	11 (21.6)	0.294
6–10	10 (13.9)	4 (19)	6 (11.8)	
11–20	18 (25)	8 (38.1)	10 (19.6)	
21–30	20 (27.8)	3 (14.3)	17 (33.3)	
>30	10 (13.9)	3 (14.3)	7 (13.7)	

* χ² test was used.

### Incidence and characteristics of workplace violence

The majority of participants (n=51; 70.8%) had either experienced or witnessed violence from patients and/or their families and friends in the previous six months. All of them reported that the violence was verbal (n=51; 100%), with no physical violence observed. No significant difference was found between the experienced violence and midwives’ age (p=0.511) or years of work experience (p=0.294). Among the 51 respondents who reported violence, 49 (96.1%) stated that the incident occurred between 1 and 20 times, while the remaining 2 midwives (3.9%) had experienced verbal violence 21–40 times. The types of verbal or non-physical violence experienced by midwives are presented in Table 2. The majority of participants (72.5%) reported facing unreasonable demands. The least common forms of verbal violence were use of social media (7.8%) and demonstrative display of anger (7.8%).

**Table 2:** Types of verbal or non-physical workplace violence experienced by midwives, as reported in the cross-sectional study conducted at the Department of Obstetrics and Gynecology of the Lithuanian University of Health Sciences, January–February 2022 (N=51)

Verbal or non-physical violence*	*n (%)*
Making unreasonable demands	37 (72.5)
Rudeness	34 (66.7)
Anger	31 (60.8)
Sarcasm	30 (58.8)
Insulting	24 (47.1)
Unjustified criticism	22 (43.1)
Berating	21 (41.2)
Swearing	20 (39.2)
Ridicule in front of others	19 (37.3)
Shouting	16 (31.4)
Gesturing	13 (25.5)
Threatening comments	11 (21.6)
Stepping into personal space	10 (19.6)
Name calling	8 (15.7)
Rumor mongering	8 (15.7)
Formal complaints without cause	8 (15.7)
Taking photographs without permission	7 (13.7)
Staring	5 (9.8)
Demonstrative display of anger	4 (7.8)
Use of social media	4 (7.8)

* Multiple responses per variable allowed.

The most often incidents of violence took place during the night hours (23:00–07:00) and on weekdays, 37.3% and 41.2% of cases, respectively (Table 3). The most frequent activity linked to episodes of violence was communication with patients and/or their relatives, friends, or visitors of patients (94.1%) followed by other activities such as providing safety measures to patients (e.g. the requirement to wear a face mask during delivery during COVID-19 pandemic) and situations where visitors were not allowed due to COVID-19 pandemic restrictions (70.6%).

**Table 3:** Episodes of verbal violence at work, by time and midwives’ activities reported by participants in the cross-sectional study conducted at the Department of Obstetrics and Gynecology of the Lithuanian University of Health Sciences, January–February 2022 (N=51)

	*n (%)*
**Time**	
7:00–15:00	10 (19.6)
15:00–23:00	8 (15.7)
23:00–7:00	19 (37.3)
Does not matter which time	14 (27.5)
**Type of day**	
Weekday	21 (41.2)
Weekend	6 (11.8)
Public holidays	5 (9.8)
Does not depend on the day	19 (37.3)
Activity*	
Communicating with patients and/or relatives, friends or visitors of patients	48 (94.1)
Triaging	26 (51.0)
Assisting patients and/or relatives, friends or visitors in waiting room	17 (33.3)
Managing reactions to delays	13 (25.5)
Positioning/turning/lifting patients	11 (21.6)
Other	36 (70.6)

* Multiple responses per variable allowed.

When asked about trends in workplace violence, 40 out of 72 respondents (55.6%) believed that the occurrence of violence episodes had remained stable, while 27 participants (37.5%) reported an increase in violence incidents at work.

### Consequences of experiencing workplace violence

Overall, 8 of 51 midwives (15.7%) reported experiencing psychological injury as a result of a violent episode. Half of them sought care from a healthcare professional, and two took time off work. The distribution of emotional and professional responses following violent episodes is shown in Table 4. Many participants reported negative emotional responses that affected their daily lives, such as altered sleep patterns, relationship issues, and symptoms of depression or low mood. Burnout or stress was reported by half of participants (50.9%) as a professional response to the violence.

**Table 4:** Emotional and professional responses of midwives following workplace violence episodes, as reported in a cross-sectional study conducted at the Department of Obstetrics and Gynecology of the Lithuanian University of Health Sciences, January–February 2022 (N=51)

Responses*	*n (%)*
**Emotional**	
Unhappiness	34 (66.7)
Anger	30 (58.8)
Irritability	21 (41.2)
Powerlessness	20 (39.2)
Altered sleep patterns	16 (31.4)
Shock/surprise	15 (29.4)
Degradation	15 (29.4)
Anxiety	13 (25.5)
Fear/anxiety for the future episodes	11 (21.6)
Relationship issues	10 (19.6)
Depression/low mood	9 (17.6)
Emotional blunting	7 (13.7)
Self-blame	7 (13.7)
Guilt	7 (13.7)
Increase in use of alcohol or other substances/medications	7 (13.7)
Panic attacks	6 (11.8)
Shame	4 (7.8)
**Professional**	
Burnout/stress	26 (50.9)
Reduced morale	18 (35.3)
Lack of empathy towards patients	14 (27.5)
Feelings of professional incompetence and self-doubt	13 (25.5)
Considered leaving current clinical area or department and moving to a lower risk unit	9 (17.7)
Considered leaving midwifery	7 (13.7)
Avoidance of patients	7 (13.7)
Decline in quality of care afforded patients	5 (9.8)
Conflict with co-workers	5 (9.8)

* Multiple responses per variable allowed.

### Actions taken after an episode of violence

The most effective actions in dealing with the psychological consequences of violence were informal debriefing with other staff members (70.6%) and talking with friends and family after an episode (52.9%). Some midwives took no action at all (17.6%) (Table 5). Two-thirds of study participants reported violent episodes: three midwives (5.9%) reported all incidents of violence, 56.9% only some incidents. One-third of study participants (37.3%) stated that they reported no incidents. Respondents were more likely not to report episodes of violence, because they did not expect anything to change in the long-term. On the other hand, none of the midwives indicated that the reporting process was too complicated or that they feared lack of support from colleagues or of being blamed for the episode. However, only 10 of the respondents were satisfied with the employer’s immediate response after the most significant episode of violence.

**Table 5:** Actions taken by midwives after an episode of workplace violence (N=51) and opinion of midwives about effective workplace violence risk prevention/minimization measures (N=72), as reported in a cross-sectional study at the Department of Obstetrics and Gynecology of the Lithuanian University of Health Sciences, January–February 2022

Responses*	*n (%)*
Effective actions in dealing with the psychological consequences of violence**	
Informal debriefing with other staff after an episode	36 (70.6)
Talking with friends and family after an episode	27 (52.9)
Took no action	9 (17.6)
Employer counselling services	4 (7.8)
Private counselling services	4 (7.8)
Factors that influence the reporting of episodes of violence**	
Don’t expect anything to change in the long-term	19 (37.2)
Lack of follow up/response from management	7 (13.7 )
Time constraints	6 (11.7 )
Too many episodes/too busy to report	6 (11.7 )
Feel you can manage these episodes effectively	6 (11.7 )
Fear of lack of support from colleagues	0 (0)
Fear of being blamed for the episode	0 (0)
It is an accepted/expected part of the job	0 (0)
Feel person was not responsible for their actions or had diminished responsibility, e.g. cognitively impaired, substance abuse, mental health issues, emotional distress	0 (0)
Process too complicated	0 (0)
Not sure how to report	0 (0)
**Measures**	
Security personnel available but based elsewhere in hospital	54 (75)
Duress alarms (hardwired and/or personal)	45 (62.5)
Use of patient management plans	39 (54.2)
Access to training not paid for by employer, e.g. course to be completed at external organization	29 (40.3)
CCTV (video surveillance)	22 (30.6)
Access to training paid for by employer, e.g. aggression minimization training	21 (29.2)
Consultation with management about prevention	20 (27.8)
Restricted access to the department, e.g. key or card access	19 (26.4)
Police called if a situation deteriorates	13 (18.1)
Signage, e.g. Zero Tolerance posters	7 (9.7)
Increased security after hours	7 (9.7)

* Multiple responses per variable allowed.

** Responses of midwives who experienced or witnessed workplace violence (N=51).

### Risk prevention strategies

The majority of midwives reported feeling secure or partly secure at work, 58.3% and 34.7%, respectively. Only 5 out of 72 (7%) indicated that they did not feel safe at work. The opinions of midwives about effective workplace violence risk prevention/minimization measures are presented in Table 5. The most common mentioned measures were security personnel (75%) and duress alarms (62.5%). Meanwhile, when answering an open question, most respondents noted that the most effective methods to prevent or minimize the incidence of violence in the workplace is training (56.9%), refusing services to violent patients (37.5%), and limiting the number of patients per healthcare provider (25%).

## DISCUSSION

Our study reveals that workplace violence against midwives is a prevalent and concerning issue within the Lithuanian perinatal healthcare setting. Specifically, 70.8% of participants reported having experienced or witnessed violence from patients or their families/friends in the past six months. The most common forms of violence were verbal/non-physical, such as unreasonable demands, rudeness and anger, while no physical violence was observed. Previous studies also indicate that workplace violence against healthcare workers is common, with one year incidence rates of verbal violence from 15.5% to 97%, followed by physical violence from 8.5% to 87%^12-21^. The discrepancy between our results and those of other studies with regard to physical violence may be explained by the different settings in which the other studies were conducted (emergency rooms, nursing homes, retirement homes)^15,21-23^ and the longer period of time during which the participants had experienced workplace violence^14,21^.

Working in a larger healthcare facility possessed a higher risk of workplace violence^23^, and our study was carried out in a perinatal center providing care for the most difficult pregnancies and births across the country. Meanwhile, older age and greater professional experience were identified as protective factors against violence and nurses exposed to violence tended to be significantly younger than those who did not experience such incidents^14,15,22^. However, midwives’ age and years of work experience did not have a significant impact on higher risk of violence in our study.

Abusive use of social media as a type of non-physical violence was reported less often (7.8%) compared to making unreasonable demands (75.2%) and rudeness (66.7%), but it showed that patients or their families/friends were willing to expand violence beyond the workplace, which could have led to more serious consequences for the midwife, such as mental or physical health problems^11,24^. The results from a global survey by Endler et al.^6^ showed that depression and insomnia were common emotional responses to workplace violence, which were also observed in our study. After the episode of violence, the most common professional response was burnout or stress. Moreover, the consequences of workplace violence in other studies were: reduced work satisfaction, decreased quality of care, and increased adverse patient related events^6,25,26^.

More than two-thirds of our study participants reported that the most effective way to deal with episodes of verbal violence was debriefing with other staff members and these findings are consistent with previous studies^12,14,16^. A small proportion of women who sought help from professional associations or nursing unions varied from 1.3% to 9.9%^12,14^. However, in our study none of the midwives mentioned that talking with a union or professional association could be an effective way to address workplace violence following an incident. This suggests that midwives’ associations may lack knowledge about reporting and support mechanisms related to workplace violence.

About one-third of midwives believed that there is an increase of violence incidents at work. The reasons for high rates of workplace violence includes staff shortages, lack of security personnel, poor communication, extended waiting periods, and unfulfilled patient expectations^6,27^. The increasing tendency of workplace violence was also observed during COVID-19 pandemic^26^. In our study, common episodes of violence occurred when enforcing the requirement to wear a face mask during delivery to prevent the spread of COVID-19 and in situations where visitors were not allowed due to COVID-19 pandemic restrictions (70.6%).

Despite the fact that two-thirds of study participants reported violent incidents, workplace violence remains underreported, bearing in mind that the rate of verbal violence ranges from 38% to 97% in Europe^20^. Another study outlined that the most common reasons for the underreporting were fear of lack of support from colleagues and lack of knowledge regarding proper reporting procedures^28^. None of the midwives in our study cited these reasons, suggesting that midwives in our hospital work within a supportive environment and that the procedures for reporting incidents of violence are well established and clearly understood. However, the fact that respondents did not report incidents of violence because they did not expect anything to change in the long-term shows that violence in the workplace is a sensitive and unresolved issue.

In our study, the majority of midwives identified security personnel as the most effective violence risk prevention and minimization measure, which is consistent with another study’s findings showing that security officers respond promptly (79.1%) and are effective in de-escalating potential violence (72.5%)^29^. Additional training in communication and conflict management skills is also considered an effective way to prevent or minimize violence^14^. However, only 8.7% of respondents in a global survey conducted by Endler et al.^6^ had received training in workplace violence. Even though training in aggression de-escalation techniques is available, no training program has yet been proven effective in preventing workplace violence^30^.

### Strengths and limitations

The present study has several limitations. This was a single-center study conducted at a specialized tertiary level care hospital that manages the most challenging pregnancies, childbirth and fetal health issues across the country. Working in such a high-risk environment could cause more stress compared to hospitals handling low-risk pregnancies. The small sample size of study participants also limits the ability to generalize these findings. A retrospective approach was used, and participants were asked to self-report on their experiences for a period of six months prior to completing the questionnaire. As a result, some events of violence may not have been accurately recalled. However, many similar studies, including those to which findings have been compared, used the same approach. Despite the limitations, this study is the first in Lithuania demonstrating the experiences of midwives with workplace violence from patients and their relatives and friends. This study highlights the significant challenges faced by midwives, emphasizing the lack of clear solutions and underreporting of incidents. It emphasizes the importance of addressing these issues and making a change. The experiences of midwives who have encountered violence in healthcare settings can help researchers and healthcare managers to better understand this phenomenon and develop reporting and support systems, as well as training programs, for the prevention and de-escalation of workplace violence. The high prevalence of verbal violence, coupled with emotional and professional consequences, demonstrates a pressing need to strengthen protective measures, training and support systems for midwives.

## CONCLUSIONS

Verbal or non-physical workplace violence affected more than two-thirds of midwives leading to significant emotional and professional distress. Although measures for reporting episodes of violence appear to be in place, a large proportion of respondents did not expect any change in the long-term, indicating that prevention policies and support of those affected are insufficient. The majority of midwives tend to rely on informal coping mechanisms. The results of the study highlight the importance of strengthening support measures and providing better training on how to deal with violence in the workplace. A survey of all midwives in Lithuania is needed to better understand the prevalence of workplace violence and the effectiveness of interventions implemented in different hospitals.

## Data Availability

The data supporting this research are available from the authors on reasonable request.
